# Treatment of polydrug-using opiate dependents during withdrawal: towards a standardisation of treatment

**DOI:** 10.1186/1471-244X-6-54

**Published:** 2006-11-15

**Authors:** Øistein Kristensen, Terje Lølandsmo, Åse Isaksen, John-Kåre Vederhus, Thomas Clausen

**Affiliations:** 1 Addiction Unit, Sørlandet Hospital, Kristiansand, Norway; 2 Unit for Addiction Medicine, Institute of Psychiatry, University of Oslo, Norway

## Abstract

**Background:**

The growing tendency among opioid addicts to misuse multiple other drugs should lead clinicians and researchers to search for new pharmacological strategies in order to prevent life-threatening complications and minimize withdrawal symptoms during polydrug detoxification.

**Methods:**

A non-randomised, open-label in-patient detoxification study was used to compare the short-time efficacy of a standardised regimen comprising 6 days Buprenorphine and 10 days Valproate (BPN/VPA) (n = 12) to a control group (n = 50) who took a 10-day traditional Clonidine/Carbamazepine (CLN/CBZ) regimen. Sixty-two dependent subjects admitted to a detoxification unit were included, all dependent on at least opioids and benzodiazepines. Other dependencies were not excluded.

**Results:**

In the BPN/VPA group, 8 out of 12 patients (67%) completed treatment compared with 25 of 50 patients (50%) in the CLN/CBZ group; this difference between the groups was non-significant (p = 0.15). Withdrawal symptoms were reduced in both groups, but only the BPN/VPA group achieved a reduction in withdrawal symptoms from day one. The difference between the two groups was significantly in favour of the BPN/VPA group for days 2 (p < 0.001), 3 (p < 0.05), 4 (p < 0.001), 5 (p < 0.01), 7 (p < 0.01) and 8 (p < 0.05). The BPN/VPA combination did not affect blood pressure, pulse or liver function, and the total burden of side-effects was experienced as modest. There appeared to be no pharmacological interactions of clinical concern, based on measurement of Buprenorphine and Valproate serum levels. Both the patients and the staff were satisfied with the standardised treatment combination.

**Conclusion:**

Overall, the combination of Buprenorphine and Valproate seems to be a safe and promising method for treating multiple drug withdrawal symptoms. The results of this study suggest that the BPN/VPA combination is potentially a better detoxification treatment for polydrug withdrawal than the traditional treatment with Clonidine and Carbamazepine. However, a randomised, double-blind study with a larger sample size to confirm our results is recommended.

**Trial registration:**

Clinical Trials.gov: NCT00367874

## Background

Withdrawal treatment appears to have no effect on long-term abstinence, so detoxification should lead to more definite drug-free treatment efforts. Nonetheless, detoxification can be a major obstacle for some patients, and the availability of managed and safe withdrawal is a prerequisite for long-term treatment.

There have been several studies on separate opioid or benzodiazepine withdrawal treatments [1-6]. However, studies that focus on polydrug detoxification are scarce, although benzodiazepine co-dependence is common in opiate abuse [7-9]. Other substances of abuse including stimulants, cannabis, hallucinogens and inhalants do not require specific treatment in patients who are being detoxified from sedative/hypnotics and/or opioids [10]. When opioid withdrawal is influenced by benzodiazepine co-dependence and withdrawal, the detoxification process becomes more complex with regard to both diagnosis and treatment regimen.

De Wet et al. report that opioid and benzodiazepine withdrawal syndromes have two distinct clinical symptoms/pathways, driven by different neurobiological mechanisms: noradrenergic activity in the locus coeruleus for opioid withdrawal and γ-aminobutyric acid (GABA-ergic) for benzodiazepine withdrawal [11]. However, there is a substantial overlap between the two in terms of clinical symptoms. These authors found that co-dependent patients concurrently detoxified from benzodiazepines and opioids reported more severe withdrawal symptoms than those detoxified from opioids alone, and concurrent benzodiazepine withdrawal exacerbated the opioid withdrawal symptoms. Benzodiazepine withdrawal diminishes the inhibitory GABA-ergic input to the locus coeruleus, hence the exacerbation.

There are traditionally two main regimens for opioid detoxification. The first uses opioid agonists such as Methadone (MET) or Buprenorphine (BPN) given in tapering doses. A meta-analysis showed no significant differences between BPN and MET in terms of retention in treatment and completers, but symptoms may resolve more quickly with BPN [12]. The second regimen uses non-opioids, medications not involved in the misuse pattern. There has been widespread use of α-adrenergic antagonists such as Clonidine (CLN) because of their ability to reduce symptoms (e.g. tremor and restlessness) due to noradrenergic hyperactivity. Treatment with CLN has been standard in Scandinavia since 1980, mainly because there has been a "system culture" of resistance to the use of opioids in withdrawal regimes. However, studies that compare BPN and CLN conclude that BPN is associated with fewer adverse effects than CLN, and completion of withdrawal treatment is significantly more likely with BPN [12,13].

Benzodiazepine withdrawal has traditionally been treated by stepwise benzodiazepine (BZD) tapering or rapid discontinuation of BZD followed by symptom-reducing medication using antiepileptics e.g. Carbamazepine (CBZ) or Valproate (VPA). Zullino et al. showed that CBZ had proved efficient in several studies [14]. However, CBZ is a cyp 3A4 inducer that interacts with opioids such as MET and BPN. An up to 60% reduction in serum Methadone level has been reported when combined with CBZ [15,16]. Interaction of CBZ with the serum BPN level has so far received less attention, but our clinical experience has shown that this combination is troublesome.

In our clinical practice we have felt the need for a standardised and safe detoxification treatment regimen for our drug addicts, as dependence on multiple drugs is so common. A standardised regimen is viewed as an improvement over the previous practice of making individual adjustments in supplementary therapy during detoxification. The overall aim of this study was to establish a new safer standard treatment without interaction problems.

VPA is theoretically a promising candidate for treating sedative-hypnotic withdrawal because of its facilitating actions on GABA levels and GABA-A receptor function [17]. VPA does not interact with opioids and has shown good results in alcohol withdrawal [18-21]. However, studies of VPA in BZD withdrawal are scarce. Research has shown that VPA 150–1200 mg/day reduced the intensity of symptoms in protracted benzodiazepine withdrawal, as well as acting as an anticonvulsant [22]. In contrast, Rickels et al. could not confirm the positive effect of VPA on BZD withdrawal symptoms [23].

The objectives of the study were 1) to assess whether a novel standardised treatment regimen – Buprenorphine (BPN) combined with Valproate (VPA) – will result in fewer withdrawal symptoms during detoxification of opiate-polydrug users than the existing treatment regimen, i.e. Clonidine (CLN) combined with Carbamazepine (CBZ); 2) to determine whether there are differences in treatment retention between the BPN/VPA and the CLN/CBZ groups; and 3) to assess differences in clinical side-effects and biochemical interactions between the two treatment regimens.

## Methods

### Setting and participants

The study was conducted in an inpatient detoxification unit. All eligible patients admitted between February and November 2003 were invited to participate. Twelve patients were included. Inclusion criteria were opiate and benzodiazepine dependence according to the ICD-10 [24]. In addition, use of opioids and benzodiazepine was verified by urine analysis. Exclusion criteria were severe mental illness, epilepsy and pregnancy. Co-dependence on other drugs (e.g. alcohol, cannabis, amphetamine or other stimulants) did not exclude individuals from participation. Mean number of dependence diagnosis per patient was 4.5 different drugs. The control group consisted of 50 patients admitted for detoxification before starting Medical-supported Maintenance Treatment (MMT) in 2000–2002; these patients met the same inclusion and exclusion criteria.

### Ethics

The study was approved by the National Committees for Research Ethics in Norway no. S-02165 and Norwegian Medicines Agency no. 02-09435. All patients in the intervention group gave written informed consent prior to treatment.

### Treatment regimens

Buprenorphine tablets (Subutex^®^) were administered sublingually for the first 6 days. The daily dosages were 6, 8, 8, 4, 2 and 2 mg, the first four days being divided into two daily doses.

Valproate (Orfiril long^®^) was given in dosage of 1200 mg, divided in two daily doses from day 3 onward. The medication was terminated when the urine was free of opiates and benzodiazepines.

The first dose of Buprenorphine was administered under supervision when the patient had obvious abstinence symptoms. The sublingual tablets were crushed to ensure good absorption and compliance.

### Control group (n = 50)

Carbamazepine (Tegretol^®^) tablets, 200 mg, 3 times daily (600 mg) for 10 days. Clonidine (Catapresan^®^) tablets, 50 – 100 μg, 3 times daily (150 – 300 μg) for 10 days.

## Measurements

### Treatment retention

The primary outcome measure for determining the success of the treatment was the proportion of patients who were retained in treatment and followed the detoxification schedule.

### Questionnaires

The *National Client Form for Addiction Treatment *[25] was administered prior to intervention to characterize the study population along sociodemographic (e.g. education, employment, social relation, housing), medical, substance use and psychiatric domains.

The *Subjective Opiate Withdrawal Scale (SOWS) *[26] contains 16 symptoms, the intensity of which the patient rates on a scale of 0 (not at all) to 4 (extremely).

*Nielsen's Benzodiazepine Withdrawal Symptom Form *[27] was created on the basis of eight European studies; it measures abstinence symptoms during benzodiazepine withdrawal. It consists of 25 items, and the patient reports the intensity of the symptoms on a scale of 0 (not at all) to 10 (extremely). The instrument is also validated to measure changes in withdrawal symptoms during treatment.

### Biochemical

#### Urine drug screening

Urine samples were collected every second day. Urine drug screening results (Rhode Diagnostika, type Micro Screen Multi Drug Screen Cup) were coded qualitatively as positive or negative for metabolites of illicit opioids (cutoff = 300 ng/ml), benzodiazepine (cutoff = 200 ng/ml), amphetamine (cutoff = 300 ng/ml and THC (cutoff = 20 ng/ml).

#### Blood analysis

Serum levels of Buprenorphine and Valproate were measured in the mornings, 12 h after administration, on days 3, 4 and 10, and analyses were conducted in the Department for Clinical Pharmacology, St. Olavs Hospital, Trondheim, using LC/MS.

### Statistical analysis

The predetermined outcome measures were retention in treatment and reduction in withdrawal symptoms assessed by various rating scales. Retention in treatment (number of days staying in treatment) was determined by Kaplan-Meier survival analysis and a log rank test. Comparison between groups for SOWS was performed using the Mann-Whitney U-test. We analysed the data using SPSS for Windows, version 12.5.

## Results

### General characteristics

Table 1 shows the background characteristics of the two groups. The control group (traditional treatment with CLN/CBZ) had the same diagnosis as the test group and had started using drugs at the same age, but was somewhat older and had a longer history of drug abuse, especially heroin and amphetamine. There were also fewer women in the control group.

**Table 1:** Demographic data of patients treated with Buprenorphine/Valproate (BPN/VPA) n = 12, and Clonidine/Carbamazepine (CLN/CBZ) n = 50.

	**BPN/VPA – group**	**CLN/CBZ – group**
Gender: Male (%)	42%	76%
Mean age (range)	28 (21–41)	38 (26–44)
Substance use:		
First time heroin use (mean age)	19	18
First time injected drugs (mean age)	20	18
Years of heroin use (mean years)	7	18
Years of BZD use (mean years)	13	19
Years of cannabis use (mean years)	14	22
Years of amphetamine use (mean years)	7	19

### Treatment retention

**Figure 1:**
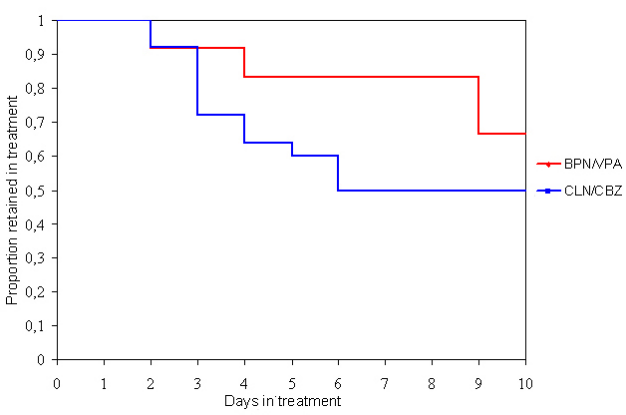
Proportion of patients retained in treatment. Kaplan-Meyer analysis. Log rank test p = 0.15. Buprenorphine/Valproate group (BPN/VPA) n = 12, and Clonidine/Carbamazepine group (CLN/CBZ) n = 50.

Figure 1 shows the proportion of patients in the two groups retained in treatment each day. Patients in the CLN/CBZ-group were less likely to complete the medical phase of detoxification than those in the BPN/VPA-group; retentions at the end were 67% (8 of 12 patients) in the BPN/VPA-group and 50% (25 of 50 patients) in the control group. The difference was not significant (p = 0.15, Kaplan-Meier survival analysis and log rank test). In the BPN/VPA group only two patients terminated treatment early. The reasons given were "craving" and restlessness. One of them was detoxified from Methadone. He had wrongly been given Buprenorphine too early, and this led to greater withdrawal distress. Two patients left the ward on day nine for unknown reasons. In the CLN/CBZ-group, 40% (20) of the patients terminated early (between days 3 and 6).

### Reduction of withdrawal symptoms

**Figure 2:**
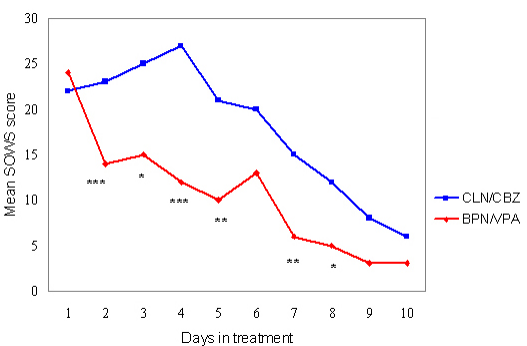
Opiate Withdrawal Symptoms during treatment with Clonidine/Carbamazepine (CLN/CBZ) n = 50, and Buprenorphine/Valproate (BPN/VPA) n = 12. Mean change in SOWS score from baseline. Lower score indicates fewer withdrawal symptoms. * p < 0.05 versus CLN/CBZ. ** p < 0.01 versus CLN/CBZ. *** p < 0.001 versus CLN/CBZ.

Figure 2 shows the mean SOWS scores for the two groups in a visual day by day comparison. Both combinations showed reduced withdrawal symptoms, but only BPN/VPA showed a reduction from the first day. The difference was significant in favour of BPN/VPA for the first four days and days seven and eight (Mann-Whitney U-test, p < 0.05). Withdrawal symptoms in the control group increased from day two to four, before the symptom burden decreased. Those who left treatment early did so during the critical period when withdrawal symptoms were most intense. About 40% had discontinued by day four in the control group with CLN/CBZ.

For the BPN/VPA group, the *Nielsen's Benzodiazepine Withdrawal Symptom Form *showed a similarly good reduction from day one, as did the SOWS score for this group. From day one to day two the average score decreased from 80 to 40 points and thereafter decreased slowly, down to 5 points on day ten.

None of the patients in either group developed psychosis or convulsions.

### Safety evaluation

**Figure 3:**
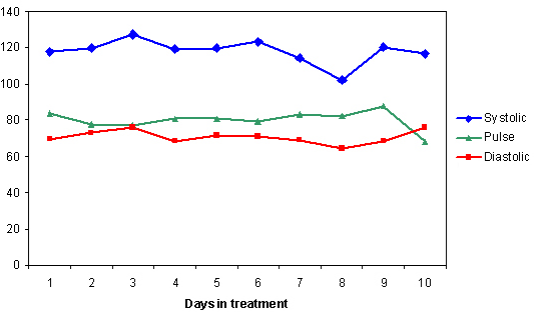
Daily measurement of blood pressure and pulse for the Buprenorphine/Valproate group (n = 12).

No serious adverse effects were reported by the BPN/VPA group and none discontinued treatment owing to adverse events. Mild discomfort from nausea was reported by two persons during the treatment phase; nausea is a well-known side-effect of Buprenorphine. Two patients in the BPN/VPA group initially mentioned headache and four reported sleep disturbance, which disappeared after two or three days. These side-effects may also be due to Buprenorphine. Sleep difficulties were treated with Alimenazin 20–40 mg or Trimipramin 25–50 mg. Drowsiness, dry mouth and fall in blood pressure were commonly reported in the CLN/CBZ group; blood pressure had to be measured every day before and after the medications were given, and the dose of Clonidine had to be regulated owing to the lowered pressure. Daily measurements in the BPN/VPA group showed that pulse and blood pressure were stable (see Fig. 3). Overall, BPN/VPA had a better adverse effect profile and was well tolerated by the patients.

### Laboratory tests

In the BPN/VPA group there was no change in haemoglobin, leucocytes or C-reactive protein (CRP) during treatment. Liver function tests (GT, AST and ALT) improved slightly (mean GT from 50 U/l to 40 U/l, mean AST from 67 U/l to 46 U/l and mean ALT from 93 U/l to 83 U/l).

### Interaction study

Measurements of serum levels of Buprenorphine alone, and the combination Buprenorphine/Valproate after steady state, showed no interaction. The mean serum level (± SD) of Buprenorphine 4 mg × 2 was 1.50 (± 0.55) nmol/l, and for the combination 1.55 (± 0.35) nmol/l. The mean serum Valproate level decreased by 26% from 512 (± 59) μmol/l for Valproate 600 mg × 2 alone to 367 (± 56) μmol/l for Valproate 600 mg × 2 combined with Buprenorphine 4 mg × 2. We think this last finding is an artefact and will offer an explanation in the discussion section.

## Discussion

The novel combination treatment, BPN/VPA, for opiate dependents with polydrug use proved to be significantly better than the previously established combination therapy, CLN/CBZ, in terms of reduced withdrawal symptoms during the observed 10-day detoxification period. Moreover, the BPN/VPA combination treatment did not involve pharmacological interactions between BPN and VPA, and generally the therapy seemed to be safe, well tolerated and free of severe side-effects. Our results also indicate, although non-significantly, that retention in treatment was improved in the BPN/VPA group, probably as a result of the diminished total burden of withdrawal symptoms especially during the first days of detoxification. An additional advantage of our suggested standardised regimen is the cost-benefit aspect. Compared to BZD withdrawal with tapering doses, as De Wet et al. described [11], our regimen reduces the time needed for withdrawal treatment.

Polydrug withdrawal treatment has scarcely been investigated in the literature. However, careful examination of the addiction monotherapy literature reveals that co-dependency with other drugs is very common. For example, in the large NIDA Clinical Trials Network that studied 13 days of Buprenorphine-Naloxone for opioid detoxification, co-dependencies with cocaine, cannabis, alcohol etc. were observed [28]. Supplementary medications were widely used to manage anxiety and restlessness (e.g. Oxazepam, Lorazepam, Phenobarbital and Hydroxyzine HCl), bone pain and arthralgias (e.g. Methocarbamol, Ibuprofen and Acetaminophen), nausea (e.g. Trimethobenzamide), diarrhoea (e.g. Loperamide and Donnatal) and insomnia (e.g. Zolpidem tartrate, Trazadone HCl, Doxepin HCl and Diphenhydramine). Most (80.3%) patients received at least one ancillary medication during the study, but the authors did not use a standard regime from the beginning.

A search of Medline and Cochrane Library using the keywords "polydrug addiction", "multiple drug addiction", "detoxification" and "withdrawal treatment" revealed only two relevant studies; both were combination studies from the same centre in Germany. Schneider et al. compared BPN/CBZ with Oxazepam/CBZ and found better retention rates in the BPN/CBZ group (73% vs. 67%) [29]. BPN/CBZ also resulted in a faster reduction of withdrawal symptoms. Seifert et al. compared BPN/CBZ with MET/CBZ and found best retention (71% vs. 42%) and proportionally fewer withdrawal symptoms the first days of treatment in the BPN/CBZ group [30]. These studies gave retention rates similar to those in our study and support our finding that BPN is the most suitable opioid agonist for treating polydrug withdrawal. However, these authors did not take into account the problem of interaction between CBZ and opioids and they did not measure serum levels, as we have done.

In our clinical practice most opioid-dependent patients abuse benzodiazepines and other drugs such as CNS stimulants and cannabis. Polydrug dependency complicates the detoxification process. Monotherapy is often not sufficient. A strict monotherapy approach (BPN or MET) also exposes patients to possible life-threatening complications such as seizures etc. The medical combination BPN/VPA has been our standard procedure since this clinical study. The patients are satisfied and so are the members of staff. For clinical practice one has to be aware of a slight increase in withdrawal symptoms on day six, representing the full breakthrough of benzodiazepine withdrawal symptoms. The symptoms decrease from day seven without supplementary medication; no one left treatment at this stage. Valproate treatment incurs some risk of birth defects (1 – 5% vs. 0,5 – 1% for Carbamazepine) [31]. We therefore recommend a pregnancy test for fertile women before the start and alternative treatment regimens for pregnant patients.

The finding of reduced serum Valproate levels requires an explanation. Buprenorphine has a half-life of 4 h; a stable serum level is achieved on day two. The blood test is taken on day three, i.e. after steady state. Both tests for Buprenorphine are therefore conducted after steady state. However, Valproate has a half-life of 16 h. Steady state requires 4–5 days of treatment. The combined medicine blood test was taken only 24 h after the first dose. Until steady state, the serum level of Valproate will increase. We therefore assume that the low serum level of Valproate measured in this combination treatment does not indicate any interaction. It rather indicates that steady state had not yet been achieved for VPA at the time of sampling. This explanation is supported by serum level measurements in a few of our patients who had a dual diagnosis of epilepsy and opioid dependence: maintenance treatment with Valproate and Buprenorphine led to no interaction with the serum level of Valproate.

### Limitations

The small number of patients included in the BPN/VPA treatment group limits the possibility of drawing firm conclusions from this study. We compared the BPN/VPA group with a historical control group of patients, and this kind of comparison introduces methodological weaknesses. For example, there may have been different motivations among the two groups. In addition, we showed that the two groups did not match perfectly in certain general characteristics (i.e. greater mean age and longer history of opiate abuse in the control group). Therefore these results must be viewed with caution and should ideally only be the precursor of a more detailed investigation, preferably a randomised controlled trial.

### Suggestions for future research

At the time of our study, it was reasonable to compare Buprenorphine with the current standard practice for the management of opioid withdrawal (CLN). However, there is now clearer evidence that Buprenorphine is more effective than Clonidine [12]. As for the adjunct medication for benzodiazepine withdrawal, Valproate was chosen due to the expectations of less pharmacological interaction with Buprenorphine.

Future research should aim to determine the additional contribution of Valproate to the management of combined opioid/benzodiazepine withdrawal. Our suggestion for appropriate comparison in future randomised controlled trials is to compare Buprenorphine alone with Buprenorphine + Valproate. Because a number of countries have Diazepam in tapering doses as a standard approach to benzodiazepine withdrawal, it would also be an alternative to include Buprenorphine + Diazepam in the comparison.

## Conclusion

The novel combination treatment, BPN/VPA, for opiate dependents with polydrug use proved to be significantly better than the previously established combination therapy, CLN/CBZ. Overall, the results indicate that BPN/VPA combination therapy is a promising, effective and safe standard treatment regimen for detoxification of opiate-dominated polydrug users. A more detailed evaluation of this new combination treatment is required before a general recommendation can be made. However, in our clinic we have already adapted this standardised BPN/VPA treatment regimen as the treatment of choice when opioid and benzodiazepine dependence has been verified.

## Competing interests

The author(s) declare that they have no competing interests.

## Authors' contributions

ØK participated in study design and interpretation, performed the analysis and drafted the manuscript.

TL and ÅI participated in study design and coordination.

JKV participated in the analysis, interpretation and drafted the manuscript.

TC participated in interpretation and drafted the manuscript.

All authors read and approved the final manuscript.

## Pre-publication history

The pre-publication history for this paper can be accessed here:


